# Editorial Notes

**Published:** 1909-03

**Authors:** 


### Editorial Notes.

**University College Colston Society.**

The Tenth Annual Dinner of this Society was held on what was last year designated as Founder's Day, January 14th, 1909, under the Presidency of the Principal of the College, Professor C. Lloyd Morgan, LL.D., F.R.S. The distinguished guest of the evening was Sir Arthur W. Rücker, M.A., D.Sc., M.D., F.R.S., late Principal of the University of London, who was supported by the Right Hon. the Lord Mayor of Bristol, Mr. Edward Robinson, J.P.

This Society was founded in 1899 for the purpose of perpetuating the memory of Edward Colston in connection with the promotion of higher education in this city, and it is now felt that the time has come when it is desirable that a portion of the funds of the Society may be used for the purpose of furthering the Bristol and West of England University scheme.

The President, in proposing "The Pious Memory of Edward Colston," said that others from that chair had laid stress upon the large sums that Edward Colston had expended in the furtherance of education, and he could not but think that Mr. George Wills's announcement a year ago from the presidential chair of the University College Colston Society of the promise of £100,000 towards the foundation of the University of Bristol would have been regarded by Edward Colston as furthering the welfare and the well-being of the citizens of Bristol. If this University of Bristol were not going to further the welfare and the well-being of the citizens of Bristol, it would certainly fail to fulfil the aims and aspirations of its promoters. He was often asked, "What will the University of Bristol be, and what will it do?" He thought the obvious answer—if in some respects a somewhat enigmatical answer—was that it would do, and would be, that which the citizens of Bristol determined that it should be and it should do. Bristol had to show the educational stuff of which it was made. It had before it a great opportunity. It had to show clearly that it was equal to the responsibilities of one of the first cities in Great Britain. No one had done more for the establishment and maintenance of Universities than the guest of the evening.

Sir Arthur Rücker, in the course of his speech, said he considered the present time to be a most interesting period in this question of University affairs in Bristol—just as the sun was rising, and had not yet risen. In his address on the subject of "University Extension," he remarked that the duties of a University might be summed up in three main points. In the first place a University had to diffuse knowledge—it had to teach. In the second place it had to add to knowledge—it had to investigate. Thirdly, it had to produce that academic spirit, that fine flower of cultivation which was so easy to recognise, but so hard to define, but was not necessarily produced either by great learning or great investigation. In all these matters the new Universities had a hard problem to solve. In teaching the rank and file of their students they had to choose between compelling them to face an examination in many of the subjects which everyone who professed to be educated must know something of, or to insist only on a more competent knowledge of a few. If they did the first they ran all the risks of fostering a system of cramming; if the second, they were sure to be accused of leaving out something which, in the opinion of enthusiasts, was of vital moment. His own opinion was that of two evils it was better to choose too few subjects than too many, and it was better to aim at teaching a man how to learn—that was, to teach himself—than to aim at teaching him, or, what was worse, examining him on, everything that everybody thought he ought to know.

Professor Rücker further spoke on the mutual interaction of the University and the city in which it was situated. He believed this connection between the University and the town would be twice blessed. It would benefit the town by directing the attention of students to the industries of the town, but it would also benefit it by establishing in its midst an outward and visible sign of its appreciation and care for things which had not always a direct commercial value, but which were as essential constituents of the greatness of a community as trade itself. Success in commerce might lead a great community to success in literature and science—had they not already had their Ramsay ?—and success in literature and science would lead to that further quickening of civil life which might again vivify and increase the commerce from which it sprang.

**Queen Victoria Jubilee Convalescent Home.** An interesting meeting was held on February 5th, under the chairmanship of Sir Herbert Ashman, for the purpose of reviewing the work of this highly successful Institution during the nine years of its life. The statistics given by the Treasurer, Mr. Philip H. Vaughan, on his 80th birthday, are of much interest, and are worthy of careful perusal.

In the nine years of the existence of the Home the average yearly receipts were £2,750, the average yearly cost was £2,558, and the average cost of food was £985. The average number of patients passing through the Home each year was 1,432, and the average number in the Home every day in the year was 67. The average stay was 17¼ days, and the average increase in weight of patients was three pounds. The total receipts were £24,766 15s. 8d., the total cost was £23,020 16s. 2d., leaving a balance of £1,745 19s. 6d. Out of that balance there was paid back to capital £400 borrowed in 1900, and, in addition to that, £1,000 was set aside for special repairs. There remained £345 19s. 6d. either in the bank or in the hands of the matron. The food cost of every patient in 1901 was £15 9s. 3d., and in 1908 £14 8s. 6d. The total cost per patient per year in 1901 was £42 12s., and in 1908 £36 10s. 3d. He thought few institutions could show a better result. The average cost per year per patient for the nine years was £38 8s. 3d. At the present time the total cost of the food per patient per month was £1 5s. 3d., or a weekly cost of 6s. 3d.

The question was raised by the Rev. Dr. Glover as to the need in Bristol of a Home for Incurables, a question demanding grave consideration and unbounded generosity.

**Home for Incurables.**

The desirability of such a Home is constantly present with those who take a part in the management of our medical institutions for the poor. Such a Home should be partially self-supporting, as it should be designed for those who are able to pay something for their maintenance. The incurable consumptive is, perhaps, the class of incurable in most urgent need of such provision. No sanatorium admits cases which are obviously beyond the range of recovery, and yet such cases should be carefully guarded against the risk of contamination of others. We cannot do otherwise than cordially commend to philanthropists the idea of endowing a Home for all classes of incurable invalids.

**Schoolboys and long runs.**

The question as to whether it is desirable or otherwise that boys of school age should be encouraged to take part in cross-country runs and races has been raised by recent correspondence appearing both in lay and medical contemporary publications, and more particularly by definite statement in a letter signed by four eminent physicians and a surgeon, "that school and cross-country races exceeding one mile in distance are wholly unsuitable for boys under the age of nineteen, as the continued strain involved is apt to cause permanent injury to the heart and other organs."

It is, of course, open to every medical man to form an opinion on such questions, and to give expression to his views; but we cannot help feeling that when a number of eminent medical men append their signatures to a statement, unless it is beyond reasonable controvertion, the grounds upon which their opinion is formed should be stated. It seems curious that cross-country runs should be regarded with suspicion by a goodly number of medical men and a larger proportion of parents, unless there is some solid basis for their objections. On the other hand, it is noteworthy that those medical men, who on such questions may be regarded as experts on the subject of exercise for boys, namely the medical officers to those Public Schools where such healthy forms of exercise are the custom, should see no reason for modifying the practice of the schools in respect to runs. Far from it: those who are best entitled to speak from particular experience appear to be unanimous in their approval of cross-country runs, and it would seem very curious if English schoolboys had to abstain from a perfectly natural form of exercise.

One naturally asks why running *a priori* should be bad for boys—should involve such a strain on the heart as has been suggested. Sprinting, which involves very rapid motion, during which breathing is very inefficiently performed, probably does throw a good deal of strain on the right heart; and in quarter or half-mile hurdle races, in which the breath is held as a rule when each hurdle is taken, this is certainly likely to put a strain on the heart. But in long races a boy has to go fairly steadily, and it is not a question of strain so much as physical endurance on the part of the central nervous system; and although a boy, if he is not in good training, may become over-exhausted, there is no reason—provided his heart is organically sound—why it should be in any way injuriously affected, even should temporary exhaustion result from undue enthusiasm on the part of the runner. Undoubtedly far greater strain to the heart may be produced by excessive gymnastic exercises, as in climbing high ropes or working on the horizontal bar. No one would blame football as a game because a boy whose heart was unsound increased the unsoundness by taking part in a game for which he was physically unfit; and although without proper medical supervision such boys would be most unsuited for cross-country races, and certainly liable to suffer from taking part in them, it would surely be absurd to try and earmark running as dangerous on account of the indiscretions of a few. Our experience, at any rate, has been that even long cross-country runs of nine miles in length may be run by boys under the age of nineteen, after proper medical supervision, without any risk of harm resulting, and we think it would be most deplorable that schoolboys should be brought up with the idea that Nature has not prepared them with necessary strength for this perfectly natural form of exercise.

**Milk.**

THE meeting of the Society on February 10th was devoted to a discussion on various points connected with milk, and abstracts of the various papers occupy a large part of this number. The papers will speak for themselves, but we have received the following "Impressionist Report of this meeting by a G.P.," whose views will receive much sympathetic consideration from our readers. He writes :—

"In an unguarded moment I wandered into this meeting to hear, as I hoped, an illuminating discussion on a topic I had thought one of the most innocent—to wit, milk. At the time of entering, our able and esteemed M.O.H. was discoursing of infant mortality (in respect of which it appears that Bristol has about the lowest record), and the influence thereon of that popular infantile drink, milk. After emphasising the importance of supervision of the distributing of that commodity, to which all heartily assented, he went on to condemn the use of raw milk unreservedly. He compared the use of it with raw meat, and insisted on the necessity of using the cooked article. Here there seemed, to me at least, ample scope for opposing opinion, *e.g.* Dr. Clement Dukes. There followed next an admirable address from our City Analyst, Dr. Russell, demonstrating the frequency of adulteration and dilution of milk, and the ridiculous inadequacy of the penalties imposed. Here again one heartily agreed, though it might be with a shudder at the possible delinquencies of one's own supplier. Then followed the *pièce de résistance* in the form of a paper from Professor Walker Hall, which was certainly one of the ablest and most strenuous addresses I have heard. It was, however, also one of the most terrorising. He demonstrated in a most convincing and masterly manner that milk, instead of being one of the blandest and most beneficial of foods, was a mixture of nameless horrors and the most virulent potentialities ; that it was blended, vended, and bought with a criminally callous or an ignorantly indifferent disregard of the most obvious precautions ; that to Pasteurise it, or to boil it (except for an hour on two consecutive days)—a task which staggers the imagination in these rushful days—was but to impair its bactericidal powers, whilst only destroying some of the moderates in the bacillic microcosm, and leaving others of the more virulent and irreconcilable free to work their wicked will. Of these and many other things of an equally unassuming character the Professor discoursed, incidentally giving the *coup de grace* to the much exploited and advertised claims of the lactic acid ferment therapy of commerce. His admirable paper made one feel one had done well to spend here a short hour of detachment from the strenuous course of outside daily life. At the same time, one felt some astonishment that so many, nourished on this death-dealing fluid, still survived to learn of its condemnation. Next spoke our venerated President, who, criticising our tendency to run sheep-like after each new theory, good or bad, devoted his short time to the task of deprecating the free use of milk, either in health or disease. In a few authoritative words he disposed of the accumulated custom and experience of all the ages, to say nothing of medical usage as obtaining during the life history of veterans like myself. Milk, it seemed, contained too much water (and the speaker was referring to the natural, unwatered product), too little of proteids, insufficient calorific material, and left too large an intestinal residue to be disposed of to be useful as a diet, except for a very short period. For elderly persons it appeared to be deadly ; for typhoid cases it was simply disastrous ; in renal cases, whether acute, subacute or chronic, its harmfulness was merely a matter of degree ; in gastric ulcer it was hardly a less death-dealing agent ; in pneumonia (and consequently in cases of heart weakness), to rely on it was only to have the unfortunate experience of him of "the pierced hand," who had leaned on the broken reed. Having shattered many of one's most cherished and firmly established beliefs as effectually as possible in the brief time at his disposal, the President gave way to his successor. Broken and dispirited, I meditated immediate flight, but temporised for a while. Then Dr. Rogers arose, and began to expound—or so it seemed to my disturbed mind—the horrors that lurked in the fluid supplied first-hand from mother to child; and I left, not standing on the order of my going. The total effect produced on one's mind, so far as it can be briefly summed up, seemed to be that to brave the perils of drinking milk is as parlous as to face the poisoned arrows of the Pigmies or the Mausers of the Boers, and requires either a profound and perhaps enviable ignorance, or a happy combination of optimism and fatalism. *In medio tutissimus ibis.*"

**Resignation of Editorial Secretary.** THE title-page of this number of our *Journal* contains a change which deserves recognition. Mr. James Taylor, who has now resigned the office of Editorial Secretary, has done ten years of steady and faithful work, and has thereby earned the gratitude of the Editorial Committee and of the Society. He has been at all times eager to further the interests of the *Journal* and the Society, and has spared himself no pains in endeavouring to improve the financial position of the *Journal*, which but for the persistent efforts of its Secretary would not be a commercial success.

The Editorial Committee is glad to welcome Dr. J. A. Nixon, who has undertaken to carry on the work of Editorial Secretary, and they trust that the members individually will facilitate his work by giving him all the assistance in their power.

